# Long-Term Use and Perceived Benefits of Goal-Oriented Attentional Self-Regulation Training in Chronic Brain Injury

**DOI:** 10.1155/2017/8379347

**Published:** 2017-02-07

**Authors:** Fred Loya, Tatjana Novakovic-Agopian, Deborah Binder, Annemarie Rossi, Scott Rome, Michelle Murphy, Anthony J.-W. Chen

**Affiliations:** ^1^San Francisco VA Medical Center, San Francisco, CA, USA; ^2^VA Northern California Health Care System, Martinez, CA, USA; ^3^University of California, San Francisco, San Francisco, CA, USA; ^4^University of California, Berkeley, Berkeley, CA, USA; ^5^California Pacific Medical Center, San Francisco, CA, USA; ^6^Laguna Honda Hospital, San Francisco, CA, USA

## Abstract

*Primary Objective.* To investigate the long-term use and perceived benefit(s) of strategies included in Goal-Oriented Attentional Self-Regulation (GOALS) training (Novakovic-Agopian et al., 2011) by individuals with acquired brain injury (ABI) and chronic executive dysfunction.* Research Design.* Longitudinal follow-up of training.* Methods and Procedures.* Sixteen participants with chronic ABI participated in structured telephone interviews 20 months (range 11 to 31 months) following completion of GOALS training. Participants responded to questions regarding the range of strategies they continued to utilize, perceived benefit(s) of strategy use, situations in which strategy use was found helpful, and functional changes attributed to training.* Results.* Nearly all participants (94%) reported continued use of at least one trained strategy in their daily lives, with 75% of participants also reporting improved functioning resulting from training. However, there was considerable variability with respect to the specific strategies individuals found helpful as well as the perceived impact of training on overall functioning.* Conclusions.* GOALS training shows promising long-term benefits for individuals in the chronic phase of brain injury. Identifying individual- and injury-level factors that account for variability in continued strategy use and the perceived long-term benefits of training will help with ongoing intervention development.

## 1. Introduction

Brain injury is a major public health concern, affecting millions of people per year at a significant societal and financial cost [1, 2]. Impaired executive control functions, especially those used to guide goal-directed functioning including attention, working memory, planning, organization, and self-monitoring, are amongst the most common and debilitating long-term consequences of brain injury [3–5]. Persistent executive dysfunction is associated with lasting disadvantage [6–9], including persistent emotional volatility, unemployment, and difficulties adapting and guiding behaviors to meet the challenges of everyday life. Training to improve metacognition [10, 11], problem solving [12], and goal management [13] has been found helpful with improving goal-directed functioning following brain injury. Indeed, such training is part of the current professional guidelines and recommendations [14, 15] for brain injury rehabilitation.

However, important questions remain with respect to whether immediate training gains can be maintained over the long term and generalized to everyday life pursuits and goals. In particular, limited information is available on the long-term functional impact of interventions designed to improve goal-directed functioning for persons with brain injury. Only a few studies contained within recent comprehensive reviews of interventions targeting goal-directed functioning [16, 17] included any follow-up data, with no studies reporting on long-term outcomes greater than six months. The few studies that have examined the long-term impact of training have generally reported positive outcomes, primarily evidenced via task-specific improvements on laboratory-based simulations [18, 19] or neuropsychological tests [20]. Although investigated to a lesser extent, previous work has also demonstrated that use of specific trained strategies can be maintained at follow-up. For instance, in a study by Fasotti et al. [18], individuals with closed head injury were taught a stepwise procedure to address deficits in information processing. At six-month follow-up, trained individuals employed more of these steps while completing simulations of everyday tasks under time pressures than control participants that received concentration training. More recently, Storzbach and colleagues [21] reported that Veterans with history of mild traumatic brain injury (TBI) who received compensatory cognitive training continued to utilize a range of trained strategies to help with planning, organization, and time management up to 5 weeks after training.

Although available data suggests that training gains may be maintained over the long term in some situations, it is often unclear in exactly which ways training continues to benefit those with brain injury. Many holistic or integrated approaches to rehabilitation [15] feature a range of strategies that target various forms and levels of dysfunction (e.g., time management, sleep problems). From a clinical perspective, it would be helpful to understand which trained strategies continue to be utilized over time, how strategy use is perceived to be beneficial, and in which situations strategies tend to be utilized. For instance, participants in Storzbach et al. [21] evidenced training benefits on a range of indicators of functioning at 5-week follow-up, including ongoing strategy use; however, it is unclear which specific strategies participants found helpful and reasons for why certain strategies were preferred over others. Subjective appraisals of these experiential factors provide critical information to guide treatment development but are often overlooked in rehabilitation research. Investigating individual experiences with training, particularly with respect to its long-term impact, may help with promoting the ongoing maintenance and generalization of strategy use to everyday goal pursuit [22, 23].

Recently, we developed an experimental, therapist-administered cognitive training protocol, Goal-Oriented Attentional Self-Regulation (GOALS) [24], for individuals with brain injury and chronic executive dysfunction (>6 months after injury). Training involves teaching the application of mindfulness-based attention regulation and goal management strategies within the context of participant-defined, real-life goals. A major emphasis of training is on improving individuals' ability to regulate their attention during different phases of goal pursuit, ranging from the initial selection of information for more in-depth processing in working memory up to task execution [25, 26]. Protecting goal-relevant information along this “goal pathway” is vital, given that goal attainment efforts can be undermined by distractions and/or disruptions at any point along the pathway. At another level, training involves instruction in goal management [13] and problem solving [12, 27], including strategies for setting clear and realistic goals, breaking them down into subgoals, and developing, monitoring, and refining action plans. This organizing framework is intended to directly help with symptoms of goal neglect [28] and to increase awareness of opportunities for use of attention regulation skills (e.g., when switching between subgoals). Training was developed based upon interventions that have been successfully applied to individuals with brain injury [12, 13, 27, 29, 30] and is hypothesized to improve self-regulatory control over neural processing of information related to all aspects of goal-directed functioning.

In an initial study [31], 16 individuals with acquired brain injuries (ABI) and chronic executive dysfunction demonstrated immediate behavioral improvements following training, relative to a brief educational comparison intervention, in targeted domains of complex attention/working memory and executive functions, as well as on a “real-world” functional task. Participants maintained the above training gains at 10-week short-term follow-up. Furthermore, they reported utilizing trained strategies to navigate challenging situations in their daily lives, even after the cessation of formal training. Participants' posttraining responses on a self-report measure indicated improvements in abilities to stop and relax during stressful times, refocus on a goal, hold goal-relevant information in mind, and break complex tasks into more manageable components. Participants further reported being able to apply some of the trained strategies to challenging situations in their lives, such as studying for and taking exams. These results provide preliminary evidence that GOALS training may help improve goal-directed functioning for individuals in the chronic phase of brain injury. However, additional follow-up is needed to assess the longer-term effects of the intervention.

Here, we provide follow-up data on these participants on the average of 20 months following training. Our primary objectives were to assess participants' continued use of trained strategies and their subjective appraisals of the long-term benefits of GOALS training. Specifically, we were interested in what aspects of training participants considered most relevant and of greatest benefit to everyday goal pursuit as well as any functional changes participants attributed to training. To explore this issue, we conducted structured interviews to ascertain the long-term impact of training on participants' lives and overall functioning.

## 2. Method

### 2.1. Participants

All 16 individuals with chronic ABI (56% female) who completed the initial study [31] participated at follow-up. Individuals were originally referred for training by their physicians and treatment providers due to history of ABI and chronic cognitive complaints involving distractibility and difficulties with problem solving, organization, and multitasking. Eleven participants had history of TBI, three had a history of stroke or cerebral hemorrhage, and one had leukoencephalopathy. Study exclusion criteria included active illicit substance use, aphasia, or any other general medical, neurologic, or psychological condition that limited participation. Mean age at study entry was 52.3 years (SD = 11.3), and time since injury ranged from six months to 23 years (median = 1.5 years). All participants remained on stable medication regimens throughout training and were independent in basic activities of daily living. The sample was highly educated (mean years of education = 16.6, SD = 1.2). Prior to injury, all participants were employed or enrolled in school full-time. At study entry, only one participant was working full-time, but in a less demanding job than prior to injury. Please refer to Novakovic-Agopian et al. [31] for more detailed description of participant characteristics.

### 2.2. Intervention and Study Design

The general theory and approach to training have been described in detail elsewhere [31]. In brief, GOALS training integrates concepts from goal management [13], problem solving [12, 27], and mindfulness training [32, 33] to address the interconnections between attentional control, working memory, and goal-directed behavior. The major emphasis of training is graded and systematic practice with strategically applying self-regulatory strategies during times when goal direction is challenged. Participants are instructed on utilizing a metacognitive strategy,* Stop-Relax-Refocus (SRR)*, which involves temporarily suspending activity, relaxing, and redirecting attention back toward the central goal when distracted, feeling overwhelmed, or otherwise taken off-task. This strategy is practiced across multiple contexts, ranging from in-session exercises to individually defined goals and settings. To facilitate* SRR* application, participants are also instructed in mindful breathing. This exercise involves keeping focused attention on the act of breathing, noticing instances of mind wandering, and redirecting attention back toward the breath when distracted. Mindful breathing is practiced in increasingly challenging situations involving distractions and/or dual-task conditions. To help guide strategic and goal-based skill application, participants are taught stepwise goal management and problem solving strategies. These strategies are practiced within the context of in-class exercises, participant-identified challenging life situations, and self-generated individual- and group-level projects.

Training consisted of ten 2-hour group sessions, three individual 1-hour sessions, and approximately 20 hours of home practice distributed over five weeks. Group size averaged three persons, and training was administered by two rehabilitation specialists with a Master's level education or greater.

### 2.3. Procedure

Participants completed a structured telephone interview lasting approximately 30 minutes, occurring an average of 20 months (range = 11–31 months) following training. Participants were asked the following open-ended questions regarding the perceived long-term impact of training on daily functioning: (i) what strategies taught during training were most useful; (ii) what aspects of daily life have been most affected by participating in training; and (iii) what goals have been achieved since training that were previously considered unattainable? We also assessed current functioning across important life domains involving work, volunteering, school, and caregiving (corresponding data had been obtained at baseline to serve as a point of comparison). Institutional Review Board approved all study procedures.

### 2.4. Data Preparation and Analysis

The content of open-ended questions was coded utilizing complementary procedures. Two study authors (FL and DB) first independently coded participants' responses based upon the primary targets of training (i.e., continued use of attention regulation and goal management strategies). Coders then met and discussed additional themes that emerged from participants' responses. These themes were retained as a unit of analysis if both authors agreed upon their conceptual relevance to the research questions (e.g., retained strategies had to be included in training, benefits had to be plausibly related to targets of training) and were endorsed by more than one participant. Coders discussed their ratings and resolved any discrepancies. Multiple category codes were permitted for each response, if applicable. Finally, the frequency of codes was tabulated and exemplars were selected to illustrate each concept.

## 3. Results

### 3.1. What Strategies or Lessons Taught Were Most Useful?

Nearly all participants (94%) reported continued use of at least one specific trained strategy in their daily lives at a minimum of 11 months following completion of GOALS training.

#### 3.1.1. Stop-Relax-Refocus (SRR) Strategy

Nearly half of the sample (*n* = 7; 44%) reported utilizing SRR strategies with the intent of regulating their attention and reducing emotional reactivity while actively pursuing their goals. This involved continued use of the entire process of Stop-Relax-Refocus (*n* = 4; 25%) as well as specific components of stop (*n* = 1; 6%), relaxation/breathing (*n* = 3; 19%), and refocusing (*n* = 1; 6%). For instance, P1 reported using SRR routinely when experiencing unanticipated distractions and/or disruptions while working toward a goal. In a similar vein, P12 reported to benefit from stopping and refocusing to manage distractions, especially while studying and working on other tasks requiring sustained concentration. P13 and P15 both reported that breathing and relaxing helped to reduce anxiety and improve focus while working on their goals.

#### 3.1.2. Goal Management

Seven participants (44%) reported ongoing use of one or more goal management strategies to assist with goal pursuit efforts. This included planning (*n* = 5), breaking down tasks into subtasks with subgoals (*n* = 2), performing daily review of previous work (*n* = 1), and improved organization, including making lists (*n* = 3). For instance, P1 reported that making detailed to-do lists helped her plan activities and stay on track while completing multistep tasks. P11 reported to make detailed plans and perform nightly reviews and checks on the day's activities, noting this routine helped him stay focused and work on his goals. P14 also noted to have greatly benefited from breaking down tasks into substeps and making plans to guide her behavior.

#### 3.1.3. Mindfulness Mediation

Three participants (19%) reported continuing to benefit from use of mindful meditation as a general practice. For instance, P7 reported ongoing practice with mindfulness meditation exercises, noting that it had an immediate calming effect and that ongoing practice improved her ability to calm down and apply SRR in challenging contexts in daily life. In addition, P12 reported benefiting from listening to mindfulness mediation recordings in situations in which she was having difficulty regulating herself.

#### 3.1.4. Other

While not included as a specific target of training per se, two participants (13%) reported that training improved their self-knowledge and self-awareness. P8 reported that the greatest lesson learned in training was “realizing her limitations,” and P1 reported that training increased her awareness of her issues.

### 3.2. What Aspects of Daily Life Have Been Most Affected by Training?

The majority of participants (*n* = 12; 75%) reported improved functioning following training. Participants' open-ended responses were categorized into broad functional domains, reflecting self-perceived benefits in areas specifically targeted by GOALS training as well as domain-general improvements. Five participants (31%) reported their ability to plan and manage goals improved, while four participants (25%) reported that they were better able to regulate their attention. For instance, P7 reported that being able to better plan aspects of daily life enabled her to persist with activities and preserve when difficulties inevitably occurred. P13 reported that training improved his ability to structure his daily life, including planning activities and utilizing a planner to stay organized. P12 reported that training resulted in an increased ability to focus for longer periods of time and to better notice instances of mind wandering or daydreaming.

Participants also reported primarily benefiting in domain-general ways via improved self-esteem (*n* = 5; 31%) and self-awareness (*n* = 6; 38%). P7 reported that training resulted in her feeling more competent with pursuing personally meaningful activities, which enabled her to accept her cognitive and functional limitations better. She attributed this acceptance to being better able to select and pursue activities that were more consistent with her perceived abilities. P10 also reported feeling less hopeless and more empowered as a result of the strategies he learned over the course of training.

Two others (13%) reported unique benefits that did not fit the above categorization, including reduced fatigue and an improved ability to socialize and communicate with friends.

### 3.3. What Goals Once Viewed as Unattainable Have Been Achieved Since Participating in Training?

The majority of participants (*n* = 10; 63%) reported that training helped them achieve important life goals they previously viewed as beyond their abilities. Specifically, four participants (40%) reported achieving goals related to work and seven participants (70%) reported increased involvement with their family and the community. For example, P9 reported utilizing trained skills to help her relearn how to drive, noting that she was better able to plan routes and felt more confident about her ability to leave the home by herself. In general, she reported that training resulted in greater mobility and less anxiety about leaving her home. P15 reported that she utilized aspects of training to help her cope with her anxiety and manage her medical issues as well as a serious and unanticipated medical condition of a loved one. This involved planning, making travel arrangements, and negotiating long commutes, tasks that she was not able to perform independently at the start of training. P11 reported that training helped her get involved with multiple activities, including participating in a community garden, singing in a church choir, and volunteering. She reported that her overall functioning and ability to handle complex tasks greatly improved following training.

### 3.4. Change to Level of Functioning over Follow-Up Period

Participants' self-reported changes across life domains involving work, volunteering, school, and caregiving following training are summarized in Table 1.

Eight participants reported being gainfully employed at some point during the follow-up period, six of whom were not working at baseline. Two participants who were employed at baseline maintained their jobs, one of whom (P1) reported that it was the longest she has worked at the same job in the 20 years since her brain injury. Of the 6 participants that returned to work, 3 were no longer working at follow-up: P3 elected to retire; P10 left her job to care for her sick husband; and P13 retired for medical reasons.

Six participants reported participating in volunteer activities at follow-up, all of whom had not been volunteering at baseline. Three participants who reported volunteering also reported making functional gains in other domains: P1 was also taking a language class for recreation, and P6 and P12 had found new employment.

Five participants reported being engaged in an educational activity during the follow-up period, only one of whom was not taking classes at baseline. P1 reported taking courses in a foreign language for recreation, a pursuit she enjoyed previously but had been unable to participate in since her injury. She reported that being able to return to school was an important personal accomplishment. In addition, four participants attending school at baseline were no longer enrolled at follow-up. Three of these participants had graduated and were gainfully employed: two had started their own businesses and one was working in the financial sector.

Five participants reported having caregiving responsibilities at follow-up, four of whom were not acting in this capacity at baseline. Of these new caregivers, P4 was the primary caregiver of a newborn; P10 had assumed primary caregiver responsibilities for an elderly parent; P11 had taken upon greater childcare responsibilities; and P15 was caring for an ill partner. Two of these participants were also engaged in other activities, including starting a small business (P4) and volunteering for multiple organizations (P11).

## 4. Discussion

The purpose of this study was to investigate the long-term impact of a research cognitive rehabilitation protocol featuring the training of strategies in goal-oriented attentional self-regulation for individuals with chronic brain injury and cognitive complaints. In particular, we were interested in participants' subjective experience of how training affected their lives in the months following the intervention, including the specific trained strategies they reported to continually utilize as well as the perceived benefit(s) associated with their application. We were also interested in any functional changes that occurred across important life domains that participants attributed to training.

Overall, a large majority of participants reported to continually use and benefit from at least one aspect of training in their daily lives. Consistent with the theoretical targets of the intervention, participants reported most frequently utilizing and benefiting from strategies to regulate attention (“Stop-Relax-Refocus”) and manage goals. Continued strategy use suggests that the training protocol was of sufficient intensity for key aspects of training to be retained and utilized over the long term without ongoing clinical support and guidance. It further suggests that training attention regulation skills within the context of participants' goals and life situations is a practical and valued approach to brain injury rehabilitation.

In addition to the specific targets of training, several participants identified deriving primary benefit via improved self-esteem and increased self-awareness. While GOALS training did not explicitly attempt to modify participants' self-perceptions, it may be that the repeated experience of applying trained strategies in their lives and achieving positive outcomes increased self-confidence and awareness of personal challenges for which strategy use might be beneficial. Increased self- efficacy in individuals recovering from brain injury has been linked with increased motivation to work toward a goal [34]. Cicerone and Azulay [35] found that perceived self-efficacy (particularly related to managing cognitive symptoms) of those with chronic TBI was the best predictor of global life satisfaction. They suggested that perceived self-efficacy for the management of cognitive symptoms may mediate the relationship between individuals' expectations and their actual achievements, thereby contributing to overall subjective well-being. Others [27, 29, 30, 36] have noted that these attitudinal, experiential, and motivational factors are critical for successful brain injury rehabilitation, recognizing that cognitive training to address objective cognitive deficits is often insufficient for yielding meaningful clinical improvements without simultaneously addressing the subjective experience of the individual. From a clinical perspective, these observations suggest there are multiple ways for rehabilitation specialists to enhance patients' self-appraisals of their abilities to manage cognitive-emotional deficits and symptoms. It is important that future research determine intervention components that increase awareness of opportunities for strategy application, as well as those that boost self-esteem and self-efficacy, such as through structuring training to provide increasingly challenging experiences to practice strategy use and ultimately develop mastery. Attending to these subjective factors may be a critical component for promoting the long-term generalization of strategies to everyday life pursuits for those with brain injury.

Participant feedback further underscored that the training experience and perceived benefits differed for individuals. Of the primary targets of training, no particular strategy emerged as consistently more valued by participants, indicating that factors unique to individuals and/or their environments may be critical for informing how a given intervention will actually be utilized by participants. Intervention studies focused solely on group-level outcomes, while highly informative, risk obfuscating findings of this nature; individuals undergoing training may benefit in distinct ways or even not at all. Elucidating patient- and injury-level factors that can help explain this variability in treatment response is needed for the continued development and refinement of cognitive training interventions.

Isolating factors that moderate treatment response will help identify individuals most likely to benefit from treatment as well as establish potential novel targets for intervention. For instance, we previously reported that pretraining parameters of brain state network organization predicted response to brain injury rehabilitation [37], signifying that individuals with certain brain network profiles may experience greater benefits from specific types of cognitive training. Findings such as this may help match individuals with treatments likely to be most helpful. They also highlight pretreatment factors that can be potentially modified to enhance training effects. One application of this approach is reflected in a two-component cognitive training protocol developed by Rath and colleagues [30]. They based their training on theory and observations that emotional self-regulation is required for successful problem solving to occur (i.e., individuals need to appropriately regulate emotional responses to problems before they can successfully implement problem solving skills). Based upon this rationale, emotional self-regulation strategies are first taught to help individuals' directly challenge negative self-efficacy beliefs prior to training problem solving skills. Spikman and colleagues [38] also found that impairments in social cognition negatively impacted training of executive functions for individuals with ABI, pointing to another potentially modifiable target that can be incorporated into a comprehensive program of brain injury rehabilitation.

In addition to treatment outcomes, it is also important to identify factors that might account for variability in strategy preference and strategy use. For instance, Evans and colleagues [39] found use of memory aids was associated with participant age, chronicity of injury, prior use of organizational systems, and attention abilities in a sample of individuals with acquired brain injury. In this study, use of memory aids was associated with greater functional independence, underscoring the importance of promoting their ongoing use as a key compensatory strategy. Attending to individual-level pretreatment factors is critical, but rarely considered in brain injury rehabilitation research.

Finally, a majority of participants reported resuming important life roles involving work, volunteering, school, and/or caregiving following training. This finding is particularly important given that participants were in the chronic phase of injury and these types of gains are often considered unlikely so long after an initial injury. Participants in this study were quite heterogeneous in their initial injuries and presentations, but all reported significant reductions in functioning across life domains that had persisted in the months and years following their injuries. While we lack evidence of direct effects of training on role resumption, these self-reported changes are encouraging. Many of our participants reported that they would not have considered taking on certain responsibilities if they had not participated in training and gained confidence in their ability to utilize trained strategies. It will be important for future studies of this and other training approaches to utilize experimental designs that include well-matched comparison interventions to help document the specificity of training effects on long-term functioning in order to draw stronger causal inferences.

These results need to be interpreted in the light of study limitations. The intervention sample was relatively small, which prevented us from examining potential individual-level factors that might moderate treatment response. In addition, the sample was highly educated and older, potentially limiting the generalizability of findings to other populations. Some of our findings (e.g., increased caregiver responsibilities following training) may be related to the age of the study sample and the roles typical amongst this cohort. It will be worthwhile to examine the long-term effects of this training approach on a more diverse sample of individuals with brain injury. Second, the time frame of long-term follow-up spanned approximately 20 months, reflecting a potentially meaningful source of variability affecting the results. It is quite plausible that the longer interval between initial study completion and follow-up contributed to some degradation in strategy recall or actual strategy use. However, we note that participants with the longest follow-up period still endorsed using at least one strategy in their daily lives. Still, future studies of skill maintenance should conform to a more standardized follow-up window to reduce this potential source of bias. Third, while we have argued that subjective appraisals and self-report are critical to ongoing treatment development, we did not include any objective indicators of treatment efficacy (e.g., performance on ecologically valid measures, neurocognitive test performance) in this study. Combining both objective and subjective indicators of the long-term impact of training may help clarify how these factors influence each other and uniquely and jointly contribute to enhanced functioning following training. This focus can help inform our understanding for what treatment components are making the most robust contribution to treatment gains. Finally, investigations of role resumption following brain injury rehabilitation would benefit from examining specific as well as general aspects of training that participants felt contributed to positive life changes. Results of the present investigation are limited to documenting the improvements themselves, but without more in-depth analysis of how, for example, use of specific strategies contributed to life changes.

In conclusion, results of this study suggest that training goal-oriented attentional self-regulation strategies may confer long-term benefit to individuals with brain injury and chronic cognitive deficits. A majority of study participants reported continuing to utilize trained strategies in their daily life as many as 31 months following training, suggesting trained strategies were highly valued and associated with substantial clinical utility. However, individuals varied in the aspects of training they found most helpful, the manner in which strategies were utilized in daily life, and the perceived benefit of strategy use. Elucidating factors that account for this variability will help further advance brain injury rehabilitation development.

## Figures and Tables

**Table 1:** Changes to functional status across life domains from baseline to follow-up.

ID	Patients' characteristics			Functional status			
	Age	Sex	Education	Work	Volunteering	School	Caregiving
*P1*	60	F	19	•	+	+	
*P2*	56	F	16		+		
*P3*	58	M	16	+		•	
*P4*	31	M	16	+		−	+
*P5*	47	M	18			•	
*P6*	34	F	16	+	+	−	
*P7*	60	F	18		+		
*P8*	63	F	16				•
*P9*	51	F	17				
*P10*	55	F	16	+			+
*P11*	45	M	18		+	•	+
*P12*	24	M	15	+	+	−	
*P13*	41	M	15	+		•	
*P14*	62	F	16			−	
*P15*	57	F	16				+
*P16*	62	M	18	•			

*Note*. •: engaged in domain at baseline and follow-up. +: engaged in domain at follow-up only. −: engaged in domain at baseline only.

## References

[1] Finkelstein E. A., Corso P. S., Miller T. R. (2006). *The Incidence and Economic Burden of Injuries in the United States*.

[2] Faul M., Xu L., Wald M. M., Coronado V. G. (2010). *Traumatic Brain Injury in the United States: Emergency Department Visits, Hospitalizations and Deaths 2002–2006*.

[3] Dean P. J. A., Sterr A. (2013). Long-term effects of mild traumatic brain injury on cognitive performance. *Frontiers in Human Neuroscience*.

[4] Lezak M. D., Howieson D. B., Loring D. W. (2004). *Neuropsychological Assessment*.

[5] Vanderploeg R. D., Curtiss G., Belanger H. G. (2005). Long-term neuropsychological outcomes following mild traumatic brain injury. *Journal of the International Neuropsychological Society*.

[6] Ponsford J. L., Downing M. G., Olver J. (2014). Longitudinal follow-up of patients with traumatic brain injury: outcome at two, five, and ten years post-injury. *Journal of Neurotrauma*.

[7] van Velzen J. M., van Bennekom C. A. M., Edelaar M. J. A., Sluiter J. K., Frings-Dresen M. H. W. (2009). How many people return to work after acquired brain injury?: a systematic review. *Brain Injury*.

[8] Zumstein M. A., Moser M., Mottini M. (2011). Long-term outcome in patients with mild traumatic brain injury: A Prospective Observational Study. *Journal of Trauma—Injury, Infection and Critical Care*.

[9] Konrad C., Geburek A. J., Rist F. (2011). Long-term cognitive and emotional consequences of mild traumatic brain injury. *Psychological Medicine*.

[10] Cheng S. K. W., Man D. W. K. (2006). Management of impaired self-awareness in persons with traumatic brain injury. *Brain Injury*.

[11] Fleming J., Ownsworth T. (2006). A review of awareness interventions in brain injury rehabilitation. *Neuropsychological Rehabilitation*.

[12] von Cramon D. Y., Cramon G. M.-V., Mai N. (1991). Problem-solving deficits in brain-injured patients: a therapeutic approach. *Neuropsychological Rehabilitation*.

[13] Levine B., Robertson I. H., Clare L. (2000). Rehabilitation of executive functioning: an experimental-clinical validation of goal management training. *Journal of the International Neuropsychological Society*.

[14] Haskins E. C., Cicerone K. D., Trexler L. E. (2012). *Cognitive Rehabilitation Manual: Translating Evidence-Based Recommendations into Practice*.

[15] Cicerone K. D., Langenbahn D. M., Braden C. (2011). Evidence-based cognitive rehabilitation: updated review of the literature from 2003 through 2008. *Archives of Physical Medicine and Rehabilitation*.

[16] Kennedy M. R. T., Coelho C., Turkstra L. (2008). Intervention for executive functions after traumatic brain injury: a systematic review, meta-analysis and clinical recommendations. *Neuropsychological Rehabilitation*.

[17] Boelen D. H. E., Spikman J. M., Fasotti L. (2011). Rehabilitation of executive disorders after brain injury: are interventions effective?. *Journal of Neuropsychology*.

[18] Fasotti L., Kovacs F., Eling P. A. T. M., Brouwer W. H. (2000). Time pressure management as a compensatory strategy training after closed head injury. *Neuropsychological Rehabilitation*.

[19] Spikman J. M., Boelen D. H. E., Lamberts K. F., Brouwer W. H., Fasotti L. (2010). Effects of a multifaceted treatment program for executive dysfunction after acquired brain injury on indications of executive functioning in daily life. *Journal of the International Neuropsychological Society*.

[20] Schweizer T. A., Levine B., Rewilak D. (2008). Rehabilitation of executive functioning after focal damage to the cerebellum. *Neurorehabilitation and Neural Repair*.

[21] Storzbach D., Twamley E. W., Roost M. S. (2016). Compensatory cognitive training for operation enduring freedom/operation iraqi freedom/operation new dawn veterans with mild traumatic brain injury. *Journal of Head Trauma Rehabilitation*.

[22] Grossman R., Salas E. (2011). The transfer of training: what really matters. *International Journal of Training and Development*.

[23] Toglia J. P. (1991). Generalization of treatment: a multicontext approach to cognitive perceptual impairment in adults with brain injury. *The American Journal of Occupational Therapy*.

[24] Novakovic-Agopian T., Rome S. R., Chen A. J. W. (2007). *Goal-Based Self-Management Training Manual*.

[25] Baddeley A. D. (2001). Is working memory still working?. *American Psychologist*.

[26] Chen A. J. W., Novakovic-Agopian T. (2012). *Interventions to Improve Cognitive Functioning After TBI*.

[27] D'Zurilla T. J., Goldfried M. R. (1971). Problem solving and behavior modification. *Journal of Abnormal Psychology*.

[28] Duncan J., Emslie H., Williams P., Johnson R., Freer C. (1996). Intelligence and the frontal lobe: the organization of goal-directed behavior. *Cognitive Psychology*.

[29] Nezu A. M., Nezu C. M., D'Zurilla T. J. (2007). *Solving Life's Problems*.

[30] Rath J. F., Simon D., Langenbahn D. M., Sherr R. L., Diller L. (2003). Group treatment of problem-solving deficits in outpatients with traumatic brain injury: a randomised outcome study. *Neuropsychological Rehabilitation*.

[31] Novakovic-Agopian T., Chen A. J.-W., Rome S. (2011). Rehabilitation of executive functioning with training in attention regulation applied to individually defined goals: a pilot study bridging theory, assessment, and treatment. *Journal of Head Trauma Rehabilitation*.

[32] Hoppes K. (2006). The application of mindfulness-based cognitive interventions in the treatment of co-occurring addictive and mood disorders. *CNS Spectrums*.

[33] McMillan T., Robertson I. H., Brock D., Chorlton L. (2002). Brief mindfulness training for attentional problems after traumatic brain injury: a randomised control treatment trial. *Neuropsychological Rehabilitation*.

[34] Wilson B. A., Gracey F., Evans J. J. (2009). *Neuropsychological Rehabilitation: Theory, Models, Therapy and Outcome*.

[35] Cicerone K. D., Azulay J. (2007). Perceived self‐efficacy and life satisfaction after traumatic brain injury. *The Journal of Head Trauma Rehabilitation*.

[36] Bandura A. (1977). Self-efficacy: toward a unifying theory of behavioral change. *Psychological Review*.

[37] Arnemann K. L., Chen A. J.-W., Novakovic-Agopian T., Gratton C., Nomura E. M., D'Esposito M. (2015). Functional brain network modularity predicts response to cognitive training after brain injury. *Neurology*.

[38] Spikman J. M., Boelen D. H. E., Pijnenborg G. H. M., Timmerman M. E., Van Der Naalt J., Fasotti L. (2013). Who benefits from treatment for executive dysfunction after brain injury? Negative effects of emotion recognition deficits. *Neuropsychological Rehabilitation*.

[39] Evans J. J., Wilson B. A., Needham P., Brentnall S. (2003). Who makes good use of memory aids? results of a survey of people with acquired brain injury. *Journal of the International Neuropsychological Society*.

